# The Sanitary Conference

**Published:** 1910-12

**Authors:** 


### BUFFALO MEDICAL JOURNAL

**A Monthly Review of Medicine and Surgery**

*EDITOR*

WILLIAM WARREN POTTER, M. D.

All communications, whether of a literary or business nature, books for review and exchanges, should be addressed to the editor, 238 DELAWARE AVENUE, BUFFALO, N.Y.

VOL. LXVI. DECEMBER, 1910. No. 5

### The Sanitary Conference.

THE tenth annual conference of the Sanitary Officers of the State of New York was held in Buffalo, November 16, 17 and 18, 1910, under the auspices of the State Department of Health. Dr. Eugene H. Porter, State Commissioner of Health, took the chair and called the first session to order at 11 A.M., introducing Dr. Francis E. Fronczak, Health Commissioner of Buffalo, who delivered a short address of welcome.

The papers read at this session related to public health and the school, and were (a) "As an Aid to Public Health Work," by John S. Wilson, of Poughkeepsie; (b) "Follow-up Work," by Franklin W. Barrows, of Buffalo; (c) "School Hygiene and School Disease," by Edward Clark, also of Buffalo; (d) "From Standpoint of Educationalist," by Thomas E. Finegan, Assistant Commissioner of Education, Albany. After discussion on these papers one was presented on "Public Health and the Dental Profession," by William G. Ebersole, of Cleveland, O., which was discussed by W. A. White, and W. W. Belcher.

At the afternoon session the first general topic was "Public Health and the Medical Profession," which was dealt with under the following subheads: (a) "Difficulties of Health Officers as Seen by the Physician," by A. D. Lake, of Gowanda; (b) "The Spirit of Helpfulness," by William D. Alsever, of Syracuse. The second general topic was "Public Health and the Press," presented under these subheads: (a) "From the Health Officer's Standpoint," by John B. Huber, of New York; (b) "From the Newspaper Man's Standpoint," by F. P. Hall, of Jamestown. These papers were discussed by Hon. Charles F. Milliken, Canandaigua, chairman State Civil Service Commission, and William H. Snyder, of Newburg. The third general topic was "Public Health and Municipal Authorities," which was presented under subheads, the first, (a) "What a Health Department Expects from Municipal Authorities," being presented by Dr. Eugene H. Porter, State Commissioner of Health. It would be well if the municipal authorities of all our cities, large and small, would familiarise themselves with this admirable presentation by Commissioner Porter.

An evening session was held at which addresses were made by C. A. Hodgetts, Ottawa, Can., whose subject was "Public Health and Conservation Movement," and by Col. Francis G. Ward, Commissioner of Public Works, Buffalo, whose subject was "Public Health and the Public Purse."

On Thursday morning sectional meetings were held, that for city health officers being presided over by Dr. Francis E. Fronczak, Health Commissioner of Buffalo, and that for rural health officers, by Dr. William A. Howe, Deputy Commissioner of Health for the State of New York. At the former section papers were read on "Garbage Disposal," by P. M. Hall, health officer, Minneapolis; "City Sanitation," by Professor Charles Baskerville, New York; and "Milk and Foods," by Dr. Fronczak. At the rural gathering a paper was read on rural hygiene and brief talks were made by the heads of divisions.

Thursday afternoon was devoted to miscellaneous matters, no formal meeting being held. In the evening a smoker was given at The Iroquois, at which there were some informal talks but no set themes or papers were offered.

At the meeting Friday the topics considered included "The Laboratory as an Aid to Diagnosis," "Reporting Communicable Disease," "Quarantine, Isolation, and Disinfection," "Control of Typhoid Fever," "Communicable Diseases, "Epidemic Anterior Poliomyelitis," and the "Tuberculosis Campaign of the State Department of Health."

It will be observed from the foregoing that the program was comprehensive and well arranged. The meeting brought together more than four hundred health officers who spent the time profitably. The moving pictures showing the danger of the housefly were very instructive, and Dr. Heath's exhibit relating to cowbarns, and his talk on efforts to obtain a pure milk supply were especially important.

The conference appears to have accomplished its purpose by bringing together a large number of health officers from the cities and towns of the state, for the discussion of questions relating to the public health. Several distinguished men from outside the state engaged in this work or interested in its thorough administration, were present and lent their valuable counsel toward its perfection, among whom, besides those already mentioned, were H. W. Hill of the epidemiological division of the Minnesota State Board of Health; Gardner T. Swarts, secretary of the Rhode Island board, and W. H. Frost of the marine hospital service, Washington, D. C.

Many local physicians and officials of the department of health were present at the several sessions, contributing in no small degree to the success of the conference, not the least of whom was Dr. Francis E. Fronczak, Health Commissioner of Buffalo.

